# ERG and OCT abnormalities in retinoblastoma after melphalan intravitreous injection

**DOI:** 10.5935/0004-2749.2021-0037

**Published:** 2022-07-04

**Authors:** Marina L. de Albuquerque, Renata Moreto, Maristella Bergamo, Zélia M. Corrêa, Rodrigo Jorge

**Affiliations:** 1 Department of Ophthalmology, Faculdade de Medicina de Ribeirão Preto, Universidade de São Paulo, Ribeirão Preto, SP, Brazil; 2 Department Pediatrics, Faculdade de Medicina de Ribeirão Preto, Universidade de São Paulo, Ribeirão Preto, SP, Brazil; 3 Departments of Ophthalmology, Bascom Palmer Eye Institute, and Sylvester Cancer Center, University of Miami, Miami, Florida, USA

**Keywords:** Retinoblastoma, Drug-related side effects and adverse reactions, Intravitreal injections, Melphalan/ toxicity, Retinoblastoma, Efeitos colaterais e reações ad­versas relacionados a medicamentos, Injeções intravítreas, Melfalan/toxicidade

## Abstract

The authors report full-field electroretinogram and optical coherence tomography findings of intravitreal melphalan retinal toxicity. An 18-month-old girl with unilateral group D retinoblastoma was evaluated with light-adapted 3 full-field electroretinogram protocol and optical coherence tomography (I-Stand optical coherence tomography, Optovue) after treatment with intravitreal melphalan for active vitreous seeds. After the third injection, the child developed retinal pigment epithelial changes near the injection site. The photopic response of the full-field electroretinogram standard flash cones showed a decrease in amplitude responses of waves a and b in the affected eye compared to the contralateral eye. Optical coherence tomography showed loss of photoreceptors and outer nuclear layers in the affected eye. Melphalan toxicity is dose-dependent, and despite its treatment benefits, it can affect vision. Our case shows an updated, in-depth retinal toxicity assessment of intravitreal melphalan in the human retina with optical coherence tomography and its correlation with electroretinogram changes.

## Abstract (pt)

Os autores relatam os achados de eletrorretinograma de campo total e tomografia de coerência óptica (OCT) da toxicidade retiniana ao melfalan intravítreo. Menina de 18 meses com retinoblastoma foi avaliada com fases fotópicas do eletrorretinograma de campo total e tomografia de coerência óptica após o tratamento com melfalan intravítreo. Após a terceira injeção, a criança desenvolveu alterações do epitélio pigmentar da retina próximo ao local da injeção. A resposta fotópica do eletrorretinograma de campo total mostrou diminuição da amplitude das respostas das ondas a e b no olho afetado comparado com o olho sadio. A tomografia de coerência óptica mostrou alterações significativas nas camadas retinianas externas no olho comprometido. A toxicidade do melfalan é dose dependente e, apesar dos benefícios terapêuticos, podem causar alterações retinianas significativas. Este caso demonstra uma avaliação atual e aprofundada da toxicidade retiniana do melfalan intravítreo na retina humana através da tomografia de coerência óptica e sua correlação com as alterações no eletrorretinograma.

## INTRODUCTION

The use of intravitreal melphalan was first introduced by Kaneko and Suzuki in the 1990s,^(1)^, and has been widely used in retinoblastoma centers all over the world after many reports demonstrated the drug efficacy and safety. This treatment emerged as a promising technique for active recurrent or persistent vitreous seeds, improving the eye salvage rate to ~87% and tumor control to ~81%^(2)^.

The control of vitreous seeds has been the main limiting factor for saving an eye with retinoblastoma, and their presence at diagnosis significantly reduces the prognosis for tumor control and eye salvage. The widespread adoption of intravitreal chemotherapy enables saving an eye that previously required enucleation^(3)^. Despite its efficacy in controlling vitreous disease, intravitreous melphalan is an alkylating agent; thus, it can cause ocular toxicity^(4)^. The posterior segment is usually involved, and electroretinogram (ERG) analysis is an important tool for objective assessment of retinal function and damage, treatment outcome, and especially, the safety of intravitreal melphalan dosage. Intravitreal melphalan injections can result in decreased ERG response, which is indicative of retinal toxicity^(5^,^6)^.

This case illustrates retinal toxicity secondary to intravitreal chemotherapy for vitreous seeds with melphalan.

## CASE REPORT

**Figure 1:**
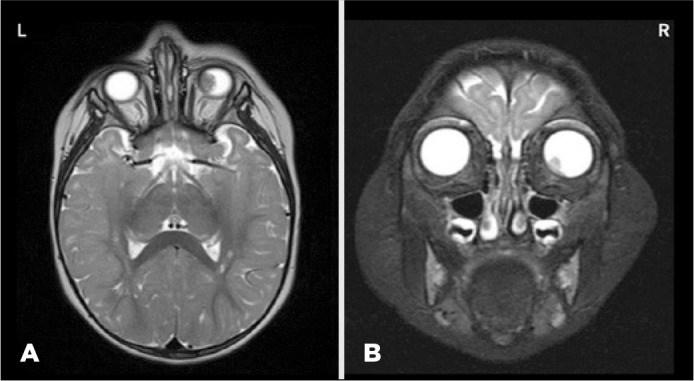
At presentation: axial [left (L)] and coronal [right (R)] magnetic resonance imaging (MRI) scan showing a hypointense mass in the left eye measuring 10.0 x 10.0 x 5.60 mm and vitreous seeding measuring 4.0 x 2.6 mm not involving the optic nerve.

An 18-month-old girl referred for evaluation of leukocoria of the left eye (OS) was submitted for ocular examination under anesthesia, brain and orbital magnetic resonance imaging (MRI), and a full physical exam by a pediatric oncologist. Under anesthesia, intraocular pressure and anterior segment were examined, and indirect ophthalmoscopy was performed. Documentation was provided with anterior segment and fundus drawings, fundus photography, posterior segment optical coherence tomography, and full-field ERG. OS showed an inferonasal solid white retinal tumor with prominent vasculature associated with an avascular dense cloud in the vitreous and prominent vitreous seeding extending to the posterior surface of the lens. MRI revealed that the tumor did not involve the optic nerve (Figure 1), and the physical exam was normal. The child was diagnosed with a group D unilateral retinoblastoma, according to International Classification for Intraocular Retinoblastoma.

**Figure 2:**
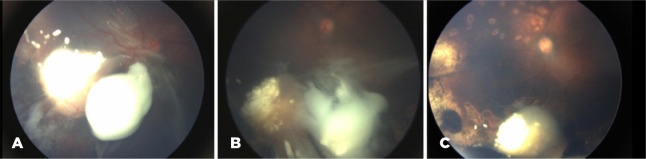
(A) After the first cycle of intravitreous chemotherapy (IVC) and immediately before the second IVC cycle, there was important tumor shrinkage associated with vitreous traction, nasal inferior retinal tear, and nasal retinal detachment (5 to 10 o’clock). There is partial regression of the nasal inferior main tumor with few calcified vitreous seeds close to it. Temporally to the main tumor, there is still an avascular vitreous mass with a noncalcified aspect. (B) After six cycles of IVC, the retina was reattached, and the main tumor presented a type three regression, but there were still active vitreous seeds (Figure 2B). (C) Color fundus picture 2 months after six cycles of IVC (vincristine, etoposide, and carboplatin) and seven injections of IVC (three melphalan and four topotecan).

The chosen treatment was single-agent intraarterial chemotherapy (IAC) with melphalan 4 mg (0.4 mg/kg)^(7)^. Four weeks later, the tumor regressed minimally, and treatment was switched to intravenous chemotherapy (IVC). The planned treatment (Brazilian protocol) consisted of six monthly cycles of IVC (vincristine, etoposide, and carboplatin) and tumor consolidation with cryotherapy. Substantial tumor shrinkage was observed after the first IVC cycle that was associated with vitreous traction, an inferonasal retinal tear, and nasal retinal detachment. Cryotherapy and laser photocoagulation were promptly performed to prevent retinal detachment progression (Figure 2A). Two months later, the retina was reattached, but there were still residual active vitreous seeds (Figure 2B), prompting additional six months of intravitreous melphalan (IVM) 30 mcg (vitreous melphalan concentration of 8 µg/ml).

The intravitreal injection was made 2.5-3.5 mm from the limbus at the desired meridian. Following the injection, the needle was withdrawn while simultaneous triple-freeze-thaw cryotherapy was delivered at the entry site. The eye was then carefully shaken in all directions for 30 seconds to achieve homogenous distribution throughout the entire vitreous cavity, and copious ocular surface irrigation was performed with sterile saline.

**Figure 3:**
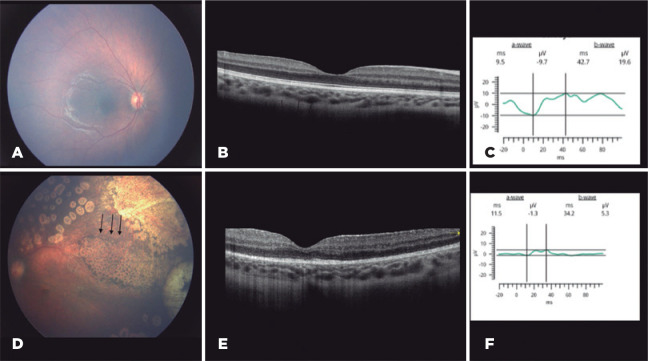
(A) Color fundus photograph of the unaffected right eye without changes on optical coherence tomography (OCT) B-scan (B) and normal amplitudes response from a- and b-waves in stimuli 3.0 cd.s/m^2^ on electroretinogram (ERG); light-adapted 3 ERG showing the a-wave amplitude of -9.7 µV and pike-time of 9.5 ms and b-wave amplitude of 19.6 µV and pike-time of 42.7 ms (C). (D) Black arrows show temporal retinal pigment epithelial mottling close to the injection site after 3 IVC with melphalan. (E) OCT B-scan of the left eye demonstrating changes to retinal pigment epithelium, photoreceptors, and ellipsoid layers. (F) ERG on the affected eye with stimuli of 3.0 cd.s/m^2^ and a b-wave reduction of 72.9% in the amplitudes responses; light-adapted 3 ERG showing the a-wave amplitude of -1.3 µV and pike-time of 11.5 ms and b-wave amplitude of 5.3 µV and pike-time of 34.2 ms.

One week after the third IVM, the patient developed retinal pigment epithelial mottling in the superotemporal retina near the injection site superotemporally (Figure 3D); thus, melphalan was discontinued. At that time, a few noncalcified vitreous seeds were still present. A decision was made to resume monthly IVC and weekly intravitreal chemotherapy with topotecan 20mcg (IVT), after which there was a complete regression of apparently active vitreous seeds (Figure 2C).

One year after the last IVT, full-field ERG (RETeval^TM^, LKC Technologies, Inc.) and optical coherence tomography (OCT B-scan) (I-Stand OCT, Optovue) were obtained during examination under anesthesia to evaluate the eye.

ERG was performed following ISCEV recommendations^(8)^ and showed a decrease in amplitudes of a- and b-waves in the photopic phase (3.0 cd.s/m^2^) (Figure 3F). Light-adapted 3 ERG recordings of retinal responses were also obtained in both eyes before 10 minutes at the light with RETeval^TM^ device and skin electrode. The skin electrodes impedance was less than 5 kΩ. The pupil diameter was 8.8 mm in the right eye and 9.3 mm in the left eye. In the right eye, light-adapted 3 ERG showed the a-wave amplitude of -9.7 µV and pike-time of 9.5 ms and b-wave amplitude of 19.6 µV and pike-time of 42.7 ms. In the left eye, light-adapted 3 ERG showed the a-wave amplitude of -1.3 µV and pike-time of 11.5 ms and b-wave amplitude of 5.3 µV and pike-time of 34.2 ms.

OCT showed retinal irregularity and a loss of photoreceptors and outer nuclear layers (Figure 3E).

Substantial tumor regression and calcification of vitreous seeding were observed following treatment despite melphalan retinal toxicity, as shown in Figure 3D.

The child has been stable, showing no signs of local tumor recurrence during the 2-years follow-up.

## DISCUSSION

Vitreous seeds are challengeable to control and a limiting factor in eye salvage due to its hypoxic state and the low chemotherapy diffusion into the vitreous^(9)^.

In our case, since there was partial regression of vitreous seeds after 3 IVM when salt and pepper reti­nopathy was detected, we decided to switch from intravitreal melphalan to topotecan. A peer literature review showed that IVT is effective in treating retinoblastoma with vitreous seeds, and its concurrent use with IVM has not demonstrated retinal toxicity thus far^(6)^.

Despite its benefits, melphalan injections can impact a patient’s visual outcome due to ocular toxicity^(7^,^9)^. Melphalan toxicity is dose-dependent, where high doses can cause retinal necrosis, vascular occlusion, and neovascularization^(1)^. Besides vascular changes, retinal damage is also due to the direct toxic effect of melphalan once in contact with the retinal surface since the vitreous tends to be thicker in childhood, and thus, the drug may be trapped by the hyaloid. The retinal pigment epithelial changes are the most common posterior segment toxicity and are generally found near the injection site with a higher drug concentration^(7)^, as seen in this case.

A usual intravitreous dose of melphalan is between 23 and 35 mcg, and it has already been demonstrated that every 30 mcg of IVM results in 5 µV degradation on ERG, which corresponds to 5% decrease for each injection^(5)^.

In addition to retinal functional loss, structural damage to the retina may occur. Such damage has only been demonstrated, until now, in rabbit eyes^(10)^.

This manuscript takes a fresh, in-depth assessment of IVM retinal toxicity in humans with OCT, and its correlation with ERG changes (Figure 3E) shows that despite the preservation of the external limiting membrane, there is loss of the ellipsoid layer and external photoreceptor segment, justifying the partially abolished ERG response shown in Figure 3F.

We chose to use the light-adapted ERG protocol recommended by ISCEV to assess the retinal function. Dark-adapted ERG was not performed due to difficulty in maintaining the darkness. The test showed a decrease in amplitude responses of a- and b-waves in the affected eye (Figure 3F) compared to the contralateral one (Figure 3C). This reduction can be justified by the damage of the cones and inner retina,^(11)^, as observed on the OCT (Figure 3E). The light-adapted 3 ERG on the affected eye showed a b-wave reduction of 72.9% in the amplitude responses.

Because the affected eye was treated with cryotherapy, IAC with melphalan, IVM, and IVT, it is unreasonable to attribute the decrease in the ERG response and OCT changes solely to IVM. It is known that cryotherapy alone leads to retinal tissue damage, and additional IAC with melphalan and IVT could have been confusing factors. However, the literature showed that IVT did not worsen ERG response,^(11^-^13)^, showing no histopathologic retinal toxicity in rabbits’ model eyes^(5)^. Besides, topotecan has a 2.5-hour intravitreal half-life after a 5-mg dose, which would not lead to accumulation during treatment^(14)^. EPR mottling has also been found following IAC with melphalan, although in our case, this only manifested after the third IVM, which concurs with published data that recognized EPR mottling usually two months after the first IVM^(15)^.

Therefore, it is reasonable to conclude that the OCT changes and decreased ERG response might have been related to IVM retinal toxicity in our retinoblastoma patient.

Intravitreal chemotherapy is an important therapeutic tool for eyes with retinoblastoma and vitreous seeding, and melphalan has proven to be effective in controlling the vitreous disease. However, additional studies are still needed to determine the range of drug concentration that is effective while minimizing retinal toxicity to allow salvaging vision and the eye.
